# The Effect of Mobile App Interventions on Influencing Healthy Maternal Behavior and Improving Perinatal Health Outcomes: Systematic Review

**DOI:** 10.2196/10012

**Published:** 2018-08-09

**Authors:** Lisa M Daly, Dell Horey, Philippa F Middleton, Frances M Boyle, Vicki Flenady

**Affiliations:** ^1^ Mater Research Institute The University of Queensland South Brisbane Australia; ^2^ School of Psychology and Public Health LaTrobe University Bundoora Australia; ^3^ Healthy Mothers, Babies and Children’s Theme South Australian Health and Medical Research Institute Adelaide Australia

**Keywords:** apps, pregnancy, perinatal, maternal, infant, mobile, systematic review, behavior change, intervention

## Abstract

**Background:**

Perinatal morbidity and mortality are significant public health issues with an enduring impact on the health and well-being of women and their families. Millions of pregnant women now download and use mobile applications to access, store, and share health information. However, little is known about the consequences. An investigation of their impact on perinatal health outcomes is particularly topical.

**Objective:**

To determine the effects of mobile app interventions during pregnancy on influencing healthy maternal behavior and improving perinatal health outcomes.

**Methods:**

Searches of PubMed, Embase, the Cochrane Library, CINAHL, WHO Global Health Library, POPLINE, and CABI Global Health were conducted with no date or language restrictions. Randomized and non-randomized studies were included if they reported perinatal health outcomes of interventions targeting pregnant women, using mobile apps compared with other communication modalities or with standard care. The primary outcome measure was the change in maternal behaviors (as defined by trial authors), by intervention goals. Two reviewers independently extracted data using standardized forms.

**Results:**

Four randomized controlled trials (RCTs) involving 456 participants were included. All studies targeted participants in early pregnancy; however, wide variation was evident in participant characteristics, intervention, and study outcomes measures. Three trials were based in hospital settings, comparing women using mobile apps with routine antenatal care. One community-based trial gave all participants a device to promote physical activity; the intervention arm was also given a mobile app. All studies reported data for the primary outcome measure, describing some benefit from the intervention compared with controls. However, few statistically significant primary or secondary outcomes were reported. Due to insufficient data, the planned meta-analysis and subgroup analyses were not performed.

**Conclusions:**

Due to limited numbers, heterogeneity of interventions, comparators, and outcome measures, no firm conclusions can be drawn on the effects of mobile application interventions during pregnancy on maternal knowledge, behavior change, and perinatal health outcomes. As millions of women utilize mobile apps during pregnancy, rigorous studies are essential for health care and maternity care providers to optimally design, implement, and evaluate interventions.

## Introduction

Adoption, practice, and maintenance of healthy behaviors during pregnancy can potentially improve maternal and child health. Adverse perinatal health outcomes such as emergency cesarean section, preterm birth, low birthweight, and stillbirth [1] are associated with maternal risk factors that may be modifiable through changes in maternal behavior [2-4]. During pregnancy and in preparation for motherhood, many women seek information and try to adopt a healthy lifestyle [5]. Pregnant women are increasingly using digital resources such as mobile apps—computer programs designed to run on mobile devices such as mobile phones and tablet computers—to access information, monitor fetal development, track individual health indicators, and provide reassurance [6-10]. Collectively, pregnancy apps have been downloaded hundreds of millions of times and are an integral source of information for many pregnant women [11]. Pregnant women may feel heightened support for informed decision-making and a sense of control by using a familiar device to access, store, and share information [9].

In 2017, over 325,000 health and fitness and medical apps were available [12]; apps directed at pregnancy constitute a major genre [13]. These apps can include health information, motivational messages, monitoring, and behavior change tools. The content of apps can be tailored by demographics such as gestational age, maternal age, language or risk factors. App developers may employ methods to engage users, such as “push communication,” including notifications designed to encourage users to follow a prompt (eg, read, listen, view content or perform an activity). Pregnancy apps may also link to a device such as a camera, glucometer, fitness activity tracker, Kegel “exerciser,” fetal heartbeat “listener,” or other monitoring equipment. Some devices associated with an app are marketed directly to consumers and avoid regulatory scrutiny, while a woman’s health care provider may provide other devices as part of clinical care.

From an institutional perspective, health systems and maternity care facilities are investigating whether and how to integrate digital patient support modalities into care and are seeking evidence to support decision making. It has been hypothesized that mobile apps may improve perinatal outcomes by encouraging access to health information, modifying demand for services, and enabling provision of targeted care [14]. This systematic review aims to determine the effects of mobile app interventions during pregnancy on influencing healthy maternal behavior and improving perinatal health outcomes, compared to interventions using other communication modalities or standard care.

## Methods

### Criteria for Considering Studies for this Review

#### Study Design

All randomized controlled trials (RCTs) and non-randomized studies including controlled before-after studies, interrupted time-series studies, and prospective comparative cohort studies were considered for inclusion. Case-control studies and cross-sectional studies were excluded due to their retrospective design. Crossover trials were also excluded as they are considered most suitable for temporary effects and chronic conditions [15]. Women at any stage of pregnancy or labor were considered for participation.

#### Interventions

Studies assessing the effects of mobile app-based interventions designed to influence maternal knowledge or behavior during pregnancy were considered for inclusion if they provided general information for pregnant women or focused on a specific maternal risk factor or perinatal outcome. There was no restriction on who sponsored the intervention or type of setting.

Studies were excluded if the intervention (1) did not utilize a mobile app, (2) used a mobile phone solely for telephone conversations or SMS (short message service) text messaging, (3) did not report on maternal or infant health outcomes, (4) did not target pregnant women (focus on clinicians, partners), and (5) focused on physical effects of mobile phone usage (such as radiation) during pregnancy.

#### Comparators

The following comparisons were planned:

1. Mobile health apps versus paper-based or SMS text messaging-based communications.
2. Mobile health apps versus interpersonal communication modes (ie, face-to-face or telephone conversation).
3. Mobile health apps versus standard care or no specified intervention.

#### Outcome Measures

The primary outcome measure was a change in maternal behaviors (as defined by trial authors), by intervention goals. Secondary outcomes addressed maternal and infant health outcomes, maternal experience with the intervention, and health service utilization measures (Table 1).

### Search Methods for Identification of Studies

#### Sources

Systematic searches were performed using seven electronic bibliographic databases: PubMed, Embase, The Cochrane Library, CINAHL, World Health Organization Global Health Library, POPLINE, and CABI Global Health. Handsearching of journals and conference proceedings from the reference lists of retrieved studies were also conducted. No language or date restrictions were applied. Abstracts and full-length articles were obtained for each citation, where available.

#### Search Strategy

The specific search strategies were developed by the primary author (LMD) and an experienced clinical research librarian, with input from all authors. Electronic searches using subject headings and all fields for keywords were conducted to avoid missing non-indexed concepts. Search terms and returns by the database and handsearching are presented in Multimedia Appendix 1. Articles published in non-peer reviewed publications were excluded, as per the review protocol. Remaining citations and abstracts were uploaded to the Web-based software platform Covidence [16]. Throughout the review process, authors were not blinded to journal titles, study authors or institutions. If it was unclear whether studies met inclusion criteria, study authors were contacted up to two times by email to request further information.

#### Study Selection

Abstracts of articles retrieved through the search strategy were independently screened by 2 review authors to determine if inclusion and exclusion criteria were met. A third author addressed any concerns about inclusion. If necessary, additional information was sought from study authors to resolve eligibility queries. For selected studies, full-text articles were obtained and read by 2 authors to confirm that they met inclusion criteria.

#### Bias Assessment

Studies were assessed for quality by 2 reviewers independently (LMD, VF), according to the guidelines in the Cochrane Handbook for Systematic Reviews of Interventions [17], by 6 domains: (1) selection bias, (2) performance bias, (3) detection bias, (4) attrition bias, (5) reporting bias, and (6) any other possible bias. All included studies were assessed on the risk of bias, likely magnitude, and potential impact on findings.

### Data Collection and Analysis

#### Data Extraction and Management

Once the studies were selected, using standardized forms, 2 reviewers (LMD, VF) independently extracted data including study objective, study design, inclusion and exclusion criteria, data sources, study period, methodology, sample size, intervention details and effects, and outcomes. Due to the complexity of the interventions found, a post-hoc decision was taken to collect data on the interventions based on the Template for Intervention Description and Replication (TIDieR) checklist [18,19].

#### Synthesis of Results

As described in the review protocol [20], we set out to synthesize data and present measures of treatment effects including summary risk ratios for dichotomous outcomes and mean difference for continuous data and subgroup analysis. Due to the considerable heterogeneity of participant characteristics, intervention features, and reported outcomes, it was decided that meta-analysis could not be performed, and results were summarized in a narrative synthesis.

**Table 1:** Primary and secondary outcome measures.

Outcome		Outcome characteristics
**Primary outcomes**		
	Maternal	Change in maternal behaviors (as defined by trial authors), by intervention goals
**Secondary outcomes**		
	Maternal	Major adverse maternal outcome (composite of death, admission to intensive care unit, or near-miss mortality as defined by the World Health Organization) Antepartum hemorrhage Postpartum hemorrhage Preeclampsia Gestational diabetes mellitus Emergency cesarean birth Successful initiation of breastfeeding Maternal knowledge (about the topic targeted by intervention) Maternal general health (as defined by standardized measures such as general health questionnaires) Maternal evaluation of the intervention (as reported by the trial) Maternal psychosocial outcomes, such as satisfaction or anxiety (as measured by any validated, standard instrument) Health service utilization (antenatal care attendance, maternal antenatal admission, length of hospital stays of mother or infant)
	Infant	Stillbirth Neonatal death Small for gestational age Large for gestational age Preterm birth (<32 weeks' gestation) Gestational age at birth Cesarean section Major neonatal morbidities (as defined by trial authors)

## Results

### Description of Studies

#### Included Studies

**Figure 1:**
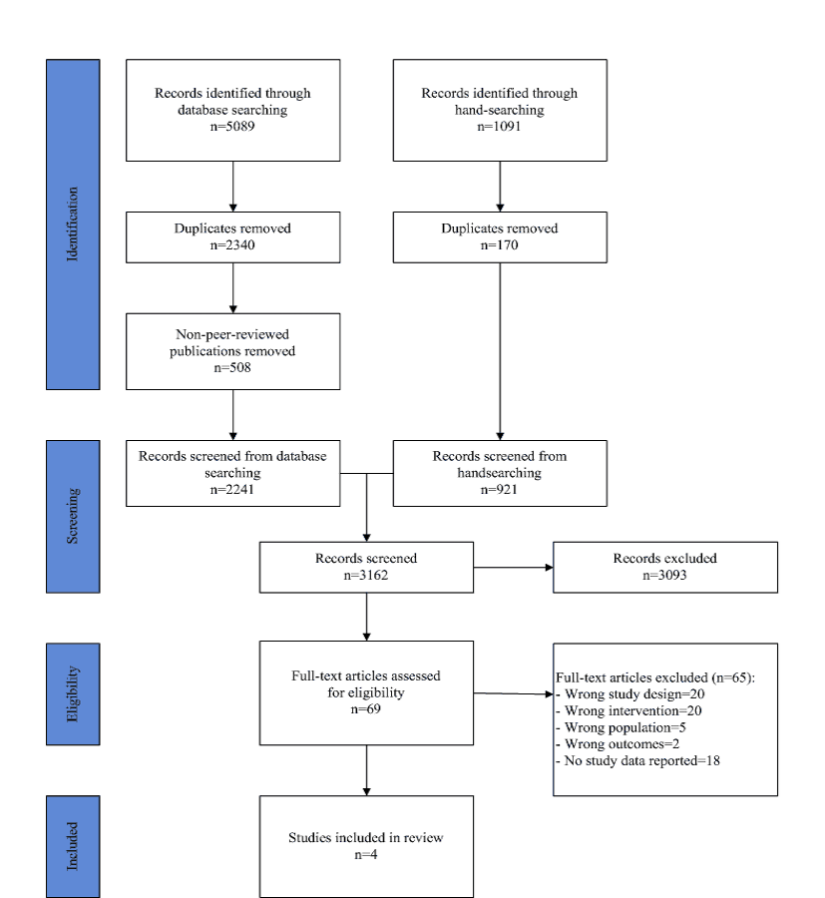
Preferred Reporting Items for Systematic Reviews and Meta-Analysis diagram of included and excluded studies.

The search strategy for this review has been consolidated into a Preferred Reporting Items for Systematic Reviews and Meta-Analysis (PRISMA) diagram (Figure 1), summarizing inclusion and exclusion of studies [21]. For database screening, one author (LMD) searched the databases on 15-16 February 2017, with a yield of 5,089 articles. After initial screening to remove duplicates and articles from non-peer reviewed journals, the titles and abstracts of 2,241 articles were reviewed by 2 authors independently (among LMD, VF, DH, PFM, and FMB). Title and abstract screening of 1,091 additional articles identified through handsearching was performed by 2 reviewers (LMD and VF); however, no additional studies were identified. Full-text screening of 69 articles was performed by 2 review authors (among LMD, VF, PFM, and DH). At all stages, disagreements between authors were resolved by consultation with a third reviewer. Reasons were recorded for excluding studies (Multimedia Appendix 2). Several articles had multiple reasons for exclusion, although each was allocated to a single category. A total of 4 trials met the inclusion criteria.

The characteristics of included studies are presented in Table 2. Though not specified as a requirement for inclusion, all studies that met inclusion criteria used RCT designs and involved pregnant women in high-income countries. Three trials were based in hospital settings, comparing women using mobile apps with standard antenatal care. One community-based trial gave all participants a device to promote physical activity; participants in the intervention arm were also given a mobile app.

#### Bias Assessment of Included Studies

Objective assessments and validated data collection tools were employed in all included studies. Studies performed generally well regarding the risk of bias in random sequence generation; 3 studies were low risk and 1 study was unclear, as it was not described. High risk of performance bias was shared across all studies. Due to the nature of mobile app interventions, blinding participants is difficult, and did not occur in any of the included studies. Blinding health care providers can also be difficult and occurred in only 1 study. Most studies had a low risk of attrition bias, with low rates of withdrawal, drop-out or loss to follow-up. Reporting biases were also low, with all studies reporting results for their respective primary outcomes. The overall risk assessment is presented in Multimedia Appendix 3.

### Description of Participants

Overall, 456 pregnant women participated in the 4 included trials, with 180 women categorized as low risk in 2 trials and 276 as moderate risk in 2 trials. Of these, 1 trial included pregnant women with asthma and another recruited pregnant women classified as overweight or obese. All trials recruited women prior to 20 weeks gestation. Table 3 shows the participant characteristics reported by each study.

### Description of Interventions

All interventions used mobile apps specifically designed for the study, rather than apps available through commercial “app stores.” None of the included studies reported if modification of the intervention occurred during the trial or described a cost-benefit analysis or compared cost of the app to other communication modalities. To motivate users to engage with the content, 2 studies developed apps utilizing “push” communication strategies. These same interventions also included a device available through a “plug-in” [23,25]. Three studies allowed users to record and track personal data within the app and provided individualized information [22,23,25]. None of the studies reported that their apps included shared participant “chat” spaces. Intervention features are described using the TIDieR checklist [18,19] presented in Table 4. Additional information about intervention characteristics—including user experience, content, patient-provider communication, functionality, and data tracking—was also collected by one reviewer (LMD) using a customized form (Multimedia Appendix 4).

### Effects of Interventions

The included trials reported different maternal and infant health outcomes such that meta-analysis or subgroup analysis was not possible.

#### Primary Outcomes

The primary outcome of interest was a change in maternal behaviors (as defined by trial authors), by intervention goals. All studies reported some type of behavior change and better results for the intervention group than controls for their respective primary outcomes (Table 5). Inventories and data collection tools used in the included studies are listed in Multimedia Appendix 5. The Ainscough et al study [22] concluded that a significantly higher proportion of the intervention group had transitioned to a “maintenance stage” of healthy lifestyle behaviors by 28 weeks’ gestation, compared to the control group (52.8% versus 32.7%, *P*=.004). The primary outcome measure for the Choi et al study [23] of physical activity was weekly mean steps, and intervention participants had a greater increase in daily steps at 12 weeks with 1096 (SD 1898) steps, compared with 259 (SD 1604) steps among control participants (*P*=.13). The change between groups reported across the 12-week study period was not statistically significant (*P*=.38). The Ledford et al study [24] found that by 32 weeks’ gestation, participants using a mobile app recorded information more frequently than the control group, and that they had developed greater “patient activation” than the control group (*F* [1127]=4.99, *P* ≥.05, n^2^=.04, marginal mean of 79.88 versus 74.81). The Zairina et al study [25] reported that the intervention group had a higher proportion of participants with well-controlled asthma than the control group (82% versus 58%, *P*=.03) at 6 months from baseline.

**Table 2:** The characteristics of included studies.

Study characteristics	Ainscough et al [22]	Choi et al [23]	Ledford et al [24]	Zairina et al [25]
Country	Ireland	United States	United States	Australia
Year	2016	2016	2016	2016
Design	Randomized controlled trial	Randomized controlled trial	Randomized controlled trial	Randomized controlled trial
Aim	To investigate the influence of a mobile phone app-supported antenatal healthy lifestyle intervention on the behavioral stage of change among overweight and obese pregnant women	To examine the feasibility of subject recruitment, randomization, intervention, and efficacy of an mHealth physical activity program for physically inactive pregnant women	To compare the effectiveness of a mobile application with a spiral-notebook guide in prenatal care	To evaluate the efficacy of a telehealth program supported by a handheld respiratory device in improving asthma control during pregnancy
Participants (risk category)	204 pregnant women with body mass index≥25 and <40 kg/m^2^ (Moderate)	30 pregnant women with a sedentary lifestyle and intent to be physically active (Low)	150 low-risk obstetrics participants following standard care pathway (Low)	72 pregnant women with asthma (Moderate)
Control group (n)	Standard care: no consistent diet or lifestyle advice offered (98)	Recommendations for gestational weight gain and safety instruction for promoting physical activity during pregnancy, and an accelerometer (15)	Standard care: a spiral notebook designed to educate participants about pregnancy and enable recording of pregnancy experiences (78)	Standard care through a participant information brochure (36)
Intervention group (n)	A “healthy lifestyle package” of individualized nutrition counseling and exercise advice, supported by a mobile phone app (106)	The same information and an accelerometer as women in the control group plus a mobile phone application, a daily message, activity diary, and feedback graphs of personal data (15)	The standard care spiral notebook replaced with a mobile app with identical content. (72)	In addition to standard care, participants were given a mobile app to record data, a proprietary medical device intended for measuring lung function (COPD-6) and a written asthma action plan (36)

**Table 3:** Participant characteristics.

Study characteristics		Ainscough et al [22]	Choi et al [23]	Ledford et al [24]	Zairina et al [25]
Participant risk category		Moderate	Low	Low	Moderate
**Total number of participants, N**		204	30	150	72
	Control, n	98	15	78	36
	Intervention, n	106	15	72	36
Inclusion characteristic among pregnant women		Body mass index≥25 and <40 kg/m^2^	Desire to increase physical activity	Low-risk	Asthma
Group differences		No	No	No	No
Age (years), mean (SD)		—^a^	33.7 (2.6)	28.91 (5.03)	31.45 (4.5)
Gestation age at recruitment (weeks); mean (SD)		15^b^	17.2 (3.4)	10-12^b^	16.35 (2.9)
Married^c^, n (%)		—	29 (97)	133 (92.4)	56 (78)
Body mass index (kg/m^2^)^d^, mean (SD)		—	27.7 (3.7)	—	28.5 (5.7)
**Race/ethnicity^e^ n (%)**					
	White	—	13 (43)	100 (69.4)	60 (83)
	Asian	—	12 (40)	8 (5.6)	6 (8)
	Black/African-American	—	2 (7)	14 (9.7)	—
	Hispanic/Latina	—	3 (10)	15 (10.4)	—
	Other	—	—	7 (4.7)	6 (8)
**Education, n (%)**					
	High school graduate	—	6 (20)	51 (34.0)	9 (13)
	University graduate	—	24 (80)	92 (61.3)	45 (63)
Ability to communicate in English		—	Yes	—	Yes

^a^Dashes indicate unreported values.

^b^Standard deviation was not reported.

^c^Studies reporting data used the term “married,” except the Choi et al study, with response category “married/cohabitating.”

^d^Choi et al reported prepregnancy body mass index. Zairina et al reported study baseline.

^e^Response categories as described by study authors.

**Table 4:** Intervention features by the Template for Intervention Description and Replication (TIDieR) checklist.

Study characteristics	Ainscough et al [22]	Choi et al [23]	Ledford et al [24]	Zairina et al [25]
Brief name (trial registration)	*Pears* (Pregnancy, exercise, and nutrition research study with mobile phone app support) study (ICTRN29316280)	*MOTHER* (Mobile Technologies to Help Enhancing Regular Physical Activity) Trial for Pregnant Women (NCT 01461707)	Mobile app as a prenatal education and engagement tool (No registration provided)	*MASTERY* (Management of Asthma with Supportive Telehealth of Respiratory function in Pregnancy Telehealth to improve asthma control in pregnancy) study (ACTRN 12613000800729)
Why	Investigate the influence of mobile app-supported antenatal healthy lifestyle intervention on the behavioral stage of change	Examine the feasibility of an mHealth physical activity program	Compare the effectiveness of a mobile app with a spiral-notebook guide in prenatal care	Evaluate the efficacy of a telehealth program supported by a handheld respiratory device in improving asthma control during pregnancy
What (materials)	Mobile app	Mobile app, accelerometer	Mobile app	Mobile app, a proprietary medical device intended for measuring lung function (COPD-6), individualized written asthma action plan (WAAP)
What (procedures)	Participants received individualized nutrition counseling and exercise advice, and mobile app. Behavioral stage-of-change score measured at baseline and late pregnancy	Participants provided with a mobile app, accelerometer, and goal-setting session at baseline. Data remotely monitored and extracted	Participants provided with a mobile app. Paper-based surveys completed at each prenatal appointment. App use assessed. Outcomes reported from health record at delivery	Participants received a COPD-6, mobile app, and individualized WAAP. Data transmitted automatically to a server accessed by researchers, participants, and clinicians. Data collected at 3 and 6 months from baseline, and after delivery
Who provided	Not described	Research staff	Antenatal care providers, researchers	Antenatal care providers, researchers
How	Mobile app	Mobile app	Mobile app	Mobile app
Where	Not described. Study authors based in Dublin, Ireland	Not described. Participants recruited by obstetricians, prenatal clinics, and communities (San Francisco, United States)	Community hospital in women’s health and family medicine departments (Maryland, United States)	Antenatal clinics of two large maternity hospitals (Melbourne, Australia)
When and how much	From (mean of) 15 weeks’ gestation to 28 weeks’ gestation	From 10-20 weeks’ gestation, continuing for 12 weeks	From 8-10 weeks’ gestation, continuing throughout pregnancy	From (mean of) 16.7 weeks’ gestation, continuing throughout pregnancy
Tailoring	Individualized nutrition and exercise advice	Individualized prescheduled weekly step goals	Not described	Individualized WAAP and weekly feedback messages
Modification of intervention during trial	Not described	Not described	Not described	Not described
Strategies to improve or maintain intervention fidelity	Not described	Feedback offered on user progress, based on uploaded activity diaries	Not described	Feedback based on individualized WAAP, lung function and asthma symptoms
Extent of intervention fidelity	Retention and adherence rates not reported	97% (29/30) participants retained during the intervention. Adherence by intervention group waned over the study period	73% (127/173) participants retained during the intervention. Change mainly due to miscarriage and elevation to high-risk care	96% (69/72) participants retained during the intervention

**Table 5:** Primary maternal outcomes: change in maternal behaviors by intervention goals.

Study	Study results	
	Primary maternal outcome	Results
Ainscough et al [22]	Shift in the stage-of-change score (transitioning from contemplation/preparation to maintenance stage of healthy lifestyle behaviors in pregnancy): baseline to 28 weeks. Study participants at Maintenance stage (stage 5).	Mean score showing a shift in stage-of-change score distribution observed for both groups. Change reported as more significant for the intervention group (*P*<.001 versus *P*=.03). The proportion in each group achieving change not reported. At 28 weeks, a higher proportion of intervention group at stage 5 (52.8%) compared with the control group (32.7%), (χ^2^=8.4, *P*=.004).
Choi et al [23]	Physical activity: change in mean steps per day.	Intervention participants had a greater increase in daily steps at 12 weeks with 1096 (SD 1898) steps, compared with 259 (SD 1604) steps among control participants (*P*=.13).
Ledford et al [24]	Patient use of a tool to find information about pregnancy (information-seeking). Patient use of tool to record information about pregnancy (information-recording). Patient activation at 32 weeks’ gestation (use of a tool).	No significant difference detected between the 2 groups (data not provided). Across all time points, intervention group recorded more frequent use of information source than the control group (*F* [1118]=4.10, *P* ≥.05, ɳ^2^=.03). The intervention group activation score was greater than controls (patient activation score marginal mean 79.88 versus 74.81 (*F* [1127]=4.99, *P* ≥.05, ɳ^2^=.04).
Zairina et al [25]	Asthma control (ACQ) 6 months from baseline.	Mean difference between groups was significant (–0.36 [SD 0.15], *P*=.02). The intervention group had higher proportion of participants with well-controlled asthma than the control group (82% versus 58%, *P*=.03).

#### Secondary Outcomes

Of the 4 studies in this review, 2 studies [23,25] report maternal secondary outcomes relevant to this review, as further detailed in Multimedia Appendix 6. One study of asthma control reports a clinically significant improvement in the asthma-related quality of life among the intervention group compared with usual care at 6 months from baseline, but the mean change was not statistically significant [25]. Another study of physical activity during pregnancy reported reduced barriers such as lack of energy, time, and willpower among the intervention group, and decreased severity of pregnancy symptoms [23]. No studies reported data on major adverse maternal outcome, maternal knowledge about the targeted health topic, maternal evaluation of the intervention, or successful initiation of breastfeeding. Furthermore, none of the trials report statistically significant differences in neonatal outcomes between intervention and control groups.

## Discussion

### Principal Findings

Despite the broad search criteria used, this systematic review identified only 4 studies for inclusion. This was an unexpected outcome of the review given the expanding use of mobile applications in maternity care. All studies included in the review reported on the primary outcome, “change in maternal behaviors by intervention goals,” but the specific outcomes reported varied by intervention. None of the studies included in this review reported statistically significant differences between intervention and control groups for neonatal outcomes, delivery or pregnancy complications. As advocated through the *Core Outcomes in Women’s and Newborn Health* initiative, a standard set of perinatal outcome measures, reported alongside those appropriate to specific health conditions or interventions, would enhance comparability [26-27]. A standardized approach using reliable and valid methods to analyze participant usage, navigation, adherence, and satisfaction would also improve comparability further and inform the design of future interventions.

Further areas for research include investigation of which intervention features yield the desired results, for example, to establish if it is an individualised clinical care plan or the advice supported by a mobile app that makes a difference. Future studies could also explore how technology can support individualized patient care plans, and if technology can be used for data tracking or streamlined reporting of symptoms to automatically prompt closer clinical scrutiny. A more in-depth exploration of the theories of behavior change underpinning study results could also add an important dimension to understand how mobile interventions influence behavior and improved perinatal outcomes.

None of the studies were designed to gauge the longitudinal benefit of mobile app interventions commenced during the perinatal period. This would be another important avenue to understand longer-term benefits, potential diminishing effects, data tracking and patient engagement opportunities offered by interventions commenced during pregnancy. Qualitative research or follow-up surveys of interventions could provide insight into users’ experiences of these interventions, including how such apps are used, and if they augment or affect perceptions of care.

Hundreds of pregnancy apps are available to the public, yet all the studies in this review developed their own mobile apps. Researchers may find it easier to guide content, facilitate communication, track data and assure user privacy with their own apps. The potential commercialization of successful interventions that could generate income for research programs could also be a consideration. However, bespoke models are likely to require more investment in development, testing, maintenance, and marketing than existing apps.

Despite creating their own apps, reviewed studies did not report extensively on their development, testing or architecture, or whether modifications were required, which would assist replication efforts. Further, none of the included studies reported an economic analysis, comparing the cost of the intervention with standard care or comparators. Policymakers and those guiding educational or clinical interventions during pregnancy would likely require such information to gauge investment alongside projected perinatal health benefits.

### Strengths and Limitations

There are several important strengths and limitations in our review. To the best of our knowledge, this systematic review is the first to assess the effects of mobile app interventions during pregnancy on influencing healthy maternal behavior and improving perinatal outcomes. This review followed an established methodology for the conduct of systematic reviews [17,19] and used a highly sensitive search strategy with no language or date restrictions to identify as many relevant studies as possible. Two authors completed the review process and extracted data independently at all stages based on prespecified criteria, and a third author participated when required to achieve consensus. Included studies were limited to those which provided comparators between mobile applications and any other intervention, including standard care, so that the role of the communication modality on intervention effects could be analyzed.

This review may have some methodological limitations. Findings are limited by the few studies that met inclusion criteria, and the small sample sizes involved in each study. Although search criteria and the databases searched were comprehensive, it is possible that relevant articles were missed. Only articles published in peer-reviewed journals were included, which may have left out some studies. Over 3,000 published study abstracts were assessed, and it was unexpected that only 4 studies would meet inclusion criteria. The heterogeneity of outcome measures further hampered the ability to meta-analyse data as originally intended, or to explore impact. Future updates of this review could search additional databases and expand the inclusion criteria to enable the analyses originally intended by the authors.

### Conclusions

As an increasing number of pregnant women use mobile apps, further research on intervention components, usage, and associated perinatal health outcomes should influence content, features, and quality of interventions. The effect of mobile app interventions on maternal knowledge, behaviour, and perinatal outcomes is still largely underreported, as evidenced by the few studies that met inclusion criteria for this review, and the minimal significant impact reported on perinatal health outcomes. Results of this systematic review may contribute to decision making by health systems, hospitals, and clinicians about the integration of mobile applications into clinical care. Emerging evidence from future trials should help to make firmer conclusions about the effectiveness of mobile app interventions during pregnancy on primary and secondary outcomes, compared to other communication modes.

## Appendix

Multimedia Appendix 1 Search terms and returns.

Multimedia Appendix 2 Excluded studies.

Multimedia Appendix 3 Bias assessment.

Multimedia Appendix 4 Intervention characteristics.

Multimedia Appendix 5 Inventories used in included studies.

Multimedia Appendix 6 Secondary outcomes.

## Abbreviations

COPD-6: a proprietary medical device intended for measuring lung function
RCT: randomized controlled trial
SMS: short message service
TIDieR: Template for Intervention Description and Replication
WAAP: written asthma action plan
