# Supplementary material for: The Effect of Mobile App Interventions on Influencing Healthy Maternal Behavior and Improving Perinatal Health Outcomes: Systematic Review
Source: JMIR Mhealth Uhealth. 2018 Aug 9;6(8):e10012. doi: 10.2196/10012 (PMC6107729; doi:10.2196/10012)
Supplement: Multimedia Appendix 1 [file mhealth_v6i8e10012_app1.pdf]

Multimedia Appendix 1: Search terms and articles retrieved

Database	Search terms	Articles retrieved	Search date	Notes
**PubMed**	Search (((((((((((("mobile app*") OR "mobile phone") OR "cell phone") OR smartphone) OR iPhone) OR iPad) OR Android) OR "handheld computer") OR "mobile applications"[MeSH Terms]) OR "smartphone"[MeSH Terms]) OR "cell phones"[MeSH Terms]) OR "computers, handheld"[MeSH Terms])) AND ((((((((((((pregnan*) OR pregnancy[MeSH Terms]) OR pregnant women[MeSH Terms]) OR matern*) OR mother*) OR prenat*) OR pre-nat) OR perinat*) OR peri-nat*) OR antenat*) OR ante-nat*) OR reproducti*)	1007	15 Feb 2017	
**Embase**	'pregnan*' OR 'matern*' OR 'mother*' OR 'prenat*' OR 'pre-nat*' OR 'perinat*' OR 'peri-nat*' OR 'antenat*' OR 'ante-nat*' OR 'reproducti*' AND ('mobile app*' OR 'mobile application' OR 'mobile phone' OR 'cell phone' OR 'smartphone' OR 'iphone' OR 'ipad' OR 'android' OR 'personal digital assistant')	1426	15 Feb 2017	All fields searched (not just .ti, .ab)
**Cochrane Library**	(pregnan* or matern* or mother* or prenat* or pre-nat* or perinat* or peri-nat* or antenat* or ante-nat* or reproducti*) and ("mobile app*" or "cell phone" or smartphone or iphone or ipad or android or handheld computer)	108	15 Feb 2017	
**CINAHL**	TX (pregnan* OR matern* OR mother* OR prenat* OR pre-nat* OR perinat* OR peri-nat* OR antenat* OR ante-nat* or reproducti*) AND TX ("mobile app" OR "mobile phone" OR "cell phone" OR smartphone OR iPhone OR iPad OR android OR handheld computer)	174	15 Feb 2017	Of these, 100 were NOT already present in Medline.
**WHO Global Health Library**	tw:((pregnan* OR matern* OR mother* OR prenat* OR pre-nat* OR perinat* OR peri-nat* OR antenat* OR ante-nat* OR reproducti*) AND ("mobile app*" OR "mobile phone" OR "cell phone" OR smartphone OR iphone OR ipad OR android OR handheld)) AND (instance:"ghl")	1697	15 Feb 2017	WHO Global Health Library only allows for title, subject, abstract lookup, not “all fields” or “all text”. 1697 returned include 1652 from Medline (duplicates).
**POPLINE**	((((pregnan* OR matern* OR mother* OR prenat* OR pre\-nat* OR perinat* OR peri\-nat* OR antenat* OR ante\-nat* OR reproducti*))) AND (((“mobile app*” OR “cell phone” OR smartphone OR iphone OR ipad OR android OR handheld computer))))) AND ((Language:English))	674	15 Feb 2017	
**CABI Global Health**	(pregnan* or matern* or mother* or prenat* or pre-nat* or perinat* or peri-nat* or antenat* or ante-nat* or reproducti*) and (“mobile app*” or “cell phone” or smartphone or iphone or ipad or android or handheld computer)	3	16 Feb 2017	To be thorough, also did simple search for “pregnan*” and “mobile*”, resulting in 201 articles, no further of relevance.
**Handsearching (post-hoc) in JMIR Publications**	(pregnan* OR perinat* OR antenat* OR prenat* OR matern* OR mother)	143	5 Apr 2018	Post-hoc handsearch in *JMIR* publications for dates up to 15 June 2017, with 143 articles returned. Two reviewers (LD & VF) screened for title/abstract and none were eligible: wrong design (56), wrong intervention (42), wrong population (45). Several articles had multiple reasons for exclusion, although each was allocated to a single category. In PRISMA diagram as “handsearching”.
**Handsearching (post-hoc) reference lists of retrieved articles**		948	6 May 2018	Post-hoc handsearch of reference lists among 69 retrieved articles (full-text assessed for eligibility), with 778 articles assessed (after duplicates removed). Ti/ab screening performed by 2 reviewers (LD & VF); none were eligible: wrong design (197), wrong intervention (463), wrong population (118). Several articles had multiple reasons for exclusion, although each was allocated to a single category. In PRISMA diagram as “handsearching”.
