# Supplementary material for: The Effect of Mobile App Interventions on Influencing Healthy Maternal Behavior and Improving Perinatal Health Outcomes: Systematic Review
Source: JMIR Mhealth Uhealth. 2018 Aug 9;6(8):e10012. doi: 10.2196/10012 (PMC6107729; doi:10.2196/10012)
Supplement: Multimedia Appendix 2 [file mhealth_v6i8e10012_app2.pdf]

Multimedia Appendix 2: Excluded studies

To assess eligibility, full-text of 69 articles was reviewed by 2 reviewers. The reason for exclusion was allocated to a single category as listed below, though several articles had multiple reasons for exclusion.

Authors (publication year)	Reason for exclusion
**Wrong study design (20)**	
Ashman, Collins et al. (2017)	No comparison arm. Intervention allows dietitian to view food photos to support paper dietary records.
Bartholomew, Church et al. (2011)	Crossover study design.
Bartholomew, Soules et al. (2015)	Crossover study design.
Daly, Baum et al. (2016)	Survey results about app usage. No comparative arm.
Fowles and Gentry (2008)	Descriptive study design.
Ganju, Krapf et al. (2016)	Compares intervention group ("low-risk") to general population statistics
García-Sáez, Martínez-Sarriegui et al. (2014)	Design evaluation of a prototype.
Hirst, Farmer et al. (2015)	Service development project; not intended to influence maternal knowledge or behaviour.
Hirst, Loerup et al. (2016)	Service development project; not intended to influence maternal knowledge or behaviour.
Hirst, Mackillop et al. (2015)	Service development project; not intended to influence maternal knowledge or behaviour.
Hirst, Mackillop et al. (2015)	Cross-sectional study design.
Knight-Agarwal, Davis et al. (2015)	Describes app development using cross-sectional and qualitative methods.
Loerup, Gibson et al. (2014)	Pilot cohort observational study; not intended to influence maternal knowledge or behaviour.
Loerup, Gibson et al. (2015)	Service development project; not intended to influence maternal knowledge or behaviour.
Loerup, Hirst et al. (2014)	Cross-sectional study design.
Mackillop, Loerup et al. (2014)	Describes development of an intervention.
Marko, Krapf et al. (2016)	Prospective observational study.
Miles and Mynard (2013)	Describes development of an app. No user or comparison data.
O'Brien, Cramp et al. (2016)	Mobile app interventions not specifically mentioned.
Tombor, Shahab et al. (2016)	About development of program.
**Wrong intervention (20)**	
Arnold, Nguyen et al. (2013)	Not a mobile app intervention.
Calle-Pascual, Pérez-Ferre et al. (2010)	SMS intervention using software to transmit data.
Colaci, Chaudhri et al. (2016)	No mobile app interventions for pregnant women mentioned.
Davis, Wambach et al. (2014)	Not a mobile app intervention. SMS and PowerPoint presentation preloaded onto an iPad.
Fawsitt, Meaney et al. (2016)	Not a mobile app intervention. Survey of women's willingness to pay for a type of intervention.
Graham, Uesugi et al. (2014)	Not a mobile app intervention.
Kingston, McDonald et al. (2014)	Not a mobile app intervention. Uses tablet for a screening questionnaire during a prenatal appointment.
Kirtley and Chien (2012)	Not a mobile app intervention. Overview of new research.
Logsdon, Davis et al. (2015)	Not a mobile app intervention; streams YouTube on iPad while mothers are in hospital.
Marcano-Belisario, Gupta et al. (2016)	Not a mobile app intervention; survey on a tablet computer.

Authors (publication year)	Reason for exclusion
Marko, Ganju et al. (2016)	As described, purpose of intervention to connect device and transmit data, not to provide information to pregnant women, influence maternal knowledge or behaviour.
Matsubara (1985)	Not a mobile app intervention.
Mauriello, Van Marter et al. (2016)	Not a mobile app intervention; web-based clinical intervention using iPad at prenatal appointments.
McLean, Osgood et al. (2017)	Feasibility study of a data collection system.
Nau (2016)	Not a mobile app intervention.
Olson, Graham et al. (2013)	Not a mobile app intervention.
Peter, Barron et al. (2015)	Not a mobile app intervention. About SMS.
Rotheram-Borus, Tomlinson et al. (2012)	Not a mobile app intervention.
Takeuchi and Horiuchi (2016)	Not a mobile app intervention; website.
Wang and Kim (2015)	Not a mobile app intervention; website.
**Wrong population (5)**	
Cheng, Huang et al. (2016)	Target population are postpartum women.
Demirci and Bogen (2016)	Target population are postpartum women.
Maycock, Scott et al. (2015)	Target population are male partners.
Ramallo-Farina, Garcia-Perez et al. (2015)	Pregnant women excluded from study.
Suleman (2015)	Target population are community midwives.
**Wrong outcomes (2)**	
Atkinson, Westeinde et al. (2016)	Immunisation focus.
Burgess, Atkinson et al. (2016)	Immunisation focus.
**No study data reported (18)**	
No named author (a) (2013)	Promotional piece about an app. No study presented.
No named author (b) (2013)	Promotional piece about an app. No study presented.
Agarwal and Labrique (2014)	Does not describe specific intervention. Studies described on SMS interventions or data management.
Choi, Fukuoka et al. (2013)	No data reported. About development of program.
Daniels and Wedler (2015)	No studies reported. About pregnant womens' app use
Garnweidner-Holme, Borgen et al. (2015)	No data reported. About app development for GDM.
Griffiths, Brown et al. (2016)	Systematic review protocol.
Kennelly, Ainscough et al. (2016)	Study protocol.
Larsson (2014)	Commentary.
Mackillop, Bartlett et al. (2016)	Study protocol.
Malloy (2015)	Commentary on radiation in utero, neurological effects
Mathew, Nimbalkar et al. (2016)	Journal club review
Mudey, Goyal et al. (2015)	No data reported. Type of intervention unclear.
Nguyen, Vu et al. (2015)	No data reported. HMIS project underway.
Schuster (2014)	Broadly describes mobile apps; not a mobile app intervention.
Stabile (2015)	Could not access article.
Stevenson, Wojcieszek et al. (2016)	Describes development of an app. No data reported.
Zairina, Abramson et al. (2015)	Study protocol.

Reference list for excluded studies (65)

(2013). "Best beginnings mobile apps: the transition to parenthood." Community Pract **86**(4): 11.

(2013). "bump buddy and baby buddy smartphone apps trialled." Nursing Children & Young People **25**(6): 5-5.

Agarwal, S. and A. Labrique (2014). "Newborn health on the line: the potential mHealth applications." Jama **312**(3): 229-230.

Arnold, C. W., T. Nguyen and C. Janzen (2013). "BabySTEPS: a sugar tracking electronic portal system for gestational diabetes." Stud Health Technol Inform **192**: 1123.

Ashman, A. M., C. E. Collins, L. J. Brown, K. M. Rae and M. E. Rollo (2017). "Validation of a Smartphone Image-Based Dietary Assessment Method for Pregnant Women." Nutrients **9**(1).

Atkinson, K. M., J. Westeinde, R. Ducharme, S. E. Wilson, S. L. Deeks, N. Crowcroft, S. Hawken and K. Wilson (2016). "Can mobile technologies improve on-time vaccination? A study piloting maternal use of ImmunizeCA, a Pan-Canadian immunization app." Human Vaccines and Immunotherapeutics **12**(10): 2654-2661.

Bartholomew, M. L., K. Church, G. Graham, J. Burlingame, I. Zalud, L. Sauvage, K. Soules and S. Shaha (2011). "Managing diabetes in pregnancy using cell phone/internet technology." American Journal of Obstetrics and Gynecology **204**(1): S113-S114.

Bartholomew, M. L., K. Soules, K. Church, S. Shaha, J. Burlingame, G. Graham, L. Sauvage and I. Zalud (2015). "Managing Diabetes in Pregnancy Using Cell Phone/Internet Technology." Clin Diabetes **33**(4): 169-174.

Burgess, K., K. M. Atkinson, J. Westeinde, N. Crowcroft, S. L. Deeks and K. Wilson (2016). "Barriers and facilitators to the use of an immunization application: a qualitative study supplemented with Google Analytics data." J Public Health (Oxf).

Calle-Pascual, A. L., N. Pérez-Ferre, M. Galindo, M. D. Fernández, V. Velasco, I. Runkle, M. J. De La Cruz, P. Martín Rojas-Marcos and L. Del Valle (2010). "The outcomes of gestational diabetes mellitus after a telecare approach are not inferior to traditional outpatient clinic visits." International Journal of Endocrinology **2010**.

Cheng, H. Y., T. Y. Huang, L. Y. Chien, Y. F. Cheng and F. J. Chen (2016). "[The Effects of a Mobile Application Social Support Program on Postpartum Perceived Stress and Depression]." Hu Li Za Zhi **63**(6): 52-60.

Choi, J., Y. Fukuoka and K. A. Lee (2013). "Development of a Mobile Phone-Based Physical Activity Program in Pregnant Women." Communicating Nursing Research **46**: 386-386.

Colaci, D., S. Chaudhri and A. Vasan (2016). "MHealth Interventions in Low-Income Countries to Address Maternal Health: A Systematic Review." Annals of Global Health.

Daly, H., A. Baum, J. Ritchie and M. Blair (2016). "Baby buddy app-a public health opportunity for new parents; evaluation of the first 46,000 downloads." Archives of Disease in Childhood **101**: A184-A185.

Daniels, M. and J. A. Wedler (2015). "Enhancing Childbirth Education through Technology." International Journal of Childbirth Education **30**(3): 28-32.

Davis, A. M., K. A. Wambach, E. L. Nelson, C. Odar, T. Lillis, A. McKinley and M. Gallagher (2014). "Health behavior change in pregnant women: a two-phase study." Telemed J E Health **20**(12): 1165-1169.

Demirci, J. R. and D. L. Bogen (2016). "Feasibility and acceptability of a mobile app in an ecological momentary assessment of early breastfeeding." Matern Child Nutr.

Fawsitt, C., S. Meaney and P. Corcoran (2016). "A cost-benefit analysis of a mobile application to monitor surgical wound post-caesarean section: A pilot-based, feasibility study." BJOG: An International Journal of Obstetrics and Gynaecology **123**: 55.

Fowles, E. R. and B. Gentry (2008). "The feasibility of personal digital assistants (PDAs) to collect dietary intake data in low-income pregnant women." J Nutr Educ Behav **40**(6): 374-377.

Ganju, N., J. Krapf, J. Benham and K. I. Marko (2016). "Preventing excessive gestational weight gain by using connected weight scales paired with mobile apps." Obstetrics and Gynecology **127**: 103S-104S.

García-Sáez, G., I. Martínez-Sarriegui, M. Rigla, B. Pons, M. Villaplana, E. J. Gómez and M. E. Hernando (2014). "Design evaluation of a prototype user interface to support a guideline-based decision support system in gestational diabetes." Diabetes Technology and Therapeutics **16**: A112.

Garnweidner-Holme, L. M., I. Borgen, I. Garitano, J. Noll and M. Lukasse (2015). "Designing and Developing a Mobile Smartphone Application for Women with Gestational Diabetes Mellitus Followed-Up at Diabetes Outpatient Clinics in Norway." Healthcare (Basel) **3**(2): 310-323.

Graham, M. L., K. H. Uesugi, J. Niederdeppe, G. K. Gay and C. M. Olson (2014). "The theory, development, and implementation of an e-intervention to prevent excessive gestational weight gain: e-Moms Roc." Telemed J E Health **20**(12): 1135-1142.

Griffiths, S. E., K. E. Brown, E. A. Fulton, I. Tombor and F. Naughton (2016). "Are digital interventions for smoking cessation in pregnancy effective? A systematic review protocol." Syst Rev **5**(1): 207.

Hirst, J. E., A. Farmer, L. Loerup, L. Mackillop, J. Levy, K. Bartlett, Y. Kenworthy, D. A. Kevat, C. Velardo and L. Tarassenko (2015). "The utility of digital blood glucose monitoring metrics to predict high birth weight in women with gestational diabetes." Diabetes **64**: A377-A378.

Hirst, J. E., L. Loerup, L. Mackillop, A. Farmer, Y. Kenworthy, K. Bartlett, C. Velardo, D. A. Kevat, L. Tarassenko and J. C. Levy (2016). "Digital blood glucose monitoring could provide new objective assessments of blood glucose control in women with gestational diabetes." Diabetic Medicine **33**(11): 1598-1599.

Hirst, J. E., L. Mackillop, L. Loerup, D. A. Kevat, K. Bartlett, O. Gibson, Y. Kenworthy, J. C. Levy, L. Tarassenko and A. Farmer (2015). "Acceptability and user satisfaction of a smartphone-based, interactive

blood glucose management system in women with gestational diabetes mellitus." J Diabetes Sci Technol **9**(1): 111-115.

Hirst, J. E., L. H. Mackillop, L. Loerup, A. J. Farmer, D. A. Kevat, K. J. Bartlett, O. J. Gibson, Y. M. Kenworthy, J. C. Levy and L. Tarassenko (2015). "GDm-health: Development of a real-time smartphone solution for the management of women with gestational diabetes mellitus (GDM)." BJOG: An International Journal of Obstetrics and Gynaecology **122**: 403.

Kennelly, M. A., K. Ainscough, K. Lindsay, E. Gibney, M. Mc Carthy and F. M. McAuliffe (2016). "Pregnancy, exercise and nutrition research study with smart phone app support (Pears): Study protocol of a randomized controlled trial." Contemp Clin Trials **46**: 92-99.

Kingston, D., S. McDonald, A. Biringer, M. P. Austin, K. Hegadoren, S. McDonald, R. Giallo, A. Ohinmaa, G. Lasiuk, G. MacQueen, W. Sword, M. Lane-Smith and S. V. van Zanten (2014). "Comparing the feasibility, acceptability, clinical-, and cost-effectiveness of mental health e-screening to paper-based screening on the detection of depression, anxiety, and psychosocial risk in pregnant women: a study protocol of a randomized, parallel-group, superiority trial." Trials **15**: 3.

Kirtley, S. and P. Chien (2012). "Women's health-what's new worldwide." BJOG: An International Journal of Obstetrics and Gynaecology **119**(3): 385-387.

Knight-Agarwal, C., D. L. Davis, L. Williams, R. Davey, R. Cox and A. Clarke (2015). "Development and Pilot Testing of the Eating4two Mobile Phone App to Monitor Gestational Weight Gain." JMIR Mhealth Uhealth **3**(2): e44.

Larsson, M. (2014). "Interventions during pregnancy and childbirth - Challenges and opportunities." Sexual and Reproductive Healthcare **5**(4): 159.

Loerup, L., O. J. Gibson, J. E. Hirst, A. J. Farmer, K. J. Bartlett, Y. M. Kenworthy, J. E. Blincowe, D. A. Kevat, L. H. MacKillop, L. Tarassenko and J. C. Levy (2014). "GDm-Health: A pilot study demonstrating the feasibility of mobile phone assisted treatment advice and medication adjustment for women with gestational diabetes." Diabetic Medicine **31**: 148-149.

Loerup, L., O. J. Gibson, J. E. Hirst, A. J. Farmer, K. J. Bartlett, Y. M. Kenworthy, D. A. Kevat, L. H. Mackillop, L. Tarassenko and J. C. Levy (2015). "A comparison of blood glucose metrics to assess the feasibility of a digital health system for management of women with gestational diabetes: The GDm-Health study." Diabetic Medicine **32**: 18-19.

Loerup, L., J. E. Hirst, L. H. MacKillop, A. Farmer, D. A. Kevat, L. Tarassenko, O. J. Gibson, K. Bartlett, Y. M. Kenworthy and J. Levy (2014). "GDm-Health: A pilot study examining acceptability of mobile phone assisted remote blood glucose monitoring for women with gestational diabetes." Diabetic Medicine **31**: 147.

Logsdon, M. C., D. Davis, D. Eckert, F. Smith, R. Stikes, J. Rushton, J. Myers, J. Capps and K. Sparks (2015). "Feasibility of Two Educational Methods for Teaching New Mothers: A Pilot Study." Interact J Med Res **4**(4): e20.

Mackillop, L., L. Loerup, K. Bartlett, A. Farmer, O. J. Gibson, J. E. Hirst, Y. Kenworthy, D. A. Kevat, J. C. Levy and L. Tarassenko (2014). "Development of a real-time smartphone solution for the management of women with or at high risk of gestational diabetes." J Diabetes Sci Technol **8**(6): 1105-1114.

Mackillop, L. H., K. Bartlett, J. Birks, A. J. Farmer, O. J. Gibson, D. A. Kevat, Y. Kenworthy, J. C. Levy, L. Loerup, L. Tarassenko, C. Velardo and J. E. Hirst (2016). "Trial protocol to compare the efficacy of a smartphone-based blood glucose management system with standard clinic care in the gestational diabetic population." BMJ Open **6**(3): e009702.

Malloy, M. E. (2015). "Babysafe Mode: Getting Smarter about Smart Technology and Pregnancy." Midwifery Today Int Midwife(116): 30-31.

Marcano-Belisario, J. S., A. K. Gupta, J. O'Donoghue, C. Morrison and J. Car (2016). "Tablet computers for implementing NICE antenatal mental health guidelines: protocol of a feasibility study." BMJ Open **6**(1): e009930.

Marko, K. I., N. Ganju, J. Brown, J. Benham and N. D. Gaba (2016). "Remote prenatal care monitoring with digital health tools can reduce visit frequency while improving satisfaction." Obstetrics and Gynecology **127**: 1S.

Marko, K. I., J. M. Krapf, A. C. Meltzer, J. Oh, N. Ganju, A. G. Martinez, S. G. Sheth and N. D. Gaba (2016). "Testing the Feasibility of Remote Patient Monitoring in Prenatal Care Using a Mobile App and Connected Devices: A Prospective Observational Trial." JMIR Res Protoc **5**(4): e200.

Mathew, J. L., S. M. Nimbalkar and V. Gopichandran (2016). "Efficacy of a mobile-based application on quality of care and perinatal mortality." Indian Pediatrics **53**(9): 823-828.

Matsubara, N. (1985). "[Studies on a handheld computer system for perinatal care]." Nihon Sanka Fujinka Gakkai Zasshi **37**(7): 1143-1150.

Mauriello, L. M., D. F. Van Marter, C. D. Umanzor, P. H. Castle and E. L. de Aguiar (2016). "Using mHealth to Deliver Behavior Change Interventions Within Prenatal Care at Community Health Centers." Am J Health Promot **30**(7): 554-562.

Maycock, B. R., J. A. Scott, Y. L. Hauck, S. K. Burns, S. Robinson, R. Giglia, A. Jorgensen, B. White, A. Harries, S. Dhaliwal, P. A. Howat and C. W. Binns (2015). "A study to prolong breastfeeding duration: design and rationale of the Parent Infant Feeding Initiative (PIFI) randomised controlled trial." BMC Pregnancy Childbirth **15**: 159.

McLean, A., N. Osgood, J. Newstead-Angel, K. Stanley, D. Knowles, W. van der Kamp, W. Qian and R. Dyck (2017). "Building Research Capacity: Results of a Feasibility Study Using a Novel mHealth Epidemiological Data Collection System Within a Gestational Diabetes Population." Stud Health Technol Inform **234**: 228-232.

Miles, M. and P. Mynard (2013). "B is for Baby: The use of iPhone app technology in childbirth education." Women & Birth **26**: S34-35.

Mudey, A., R. Goyal, G. Dangre-Mudey and M. Khapre (2015). "Tracking of antenatal mothers and neonates in rural and remote area by mobile e-health model: An innovative approach." European Journal of Epidemiology **30**(8): 867.

Nau, J. Y. (2016). "[Not Available]." Rev Med Suisse **12**(518): 958-959.

Nguyen, L. T., N. Vu, H. V. Duong, J. O'Neil, D. Wiljer and C. K. Nguyen (2015). "mMOM - Improving maternal and child health for ethnic minority people in mountainous region of Thai Nguyen province of Vietnam through integration of mHealth in HMIS and user-provider interaction." Annals of Global Health **81**(1): 195-196.

O'Brien, C. M., C. Cramp and J. M. Dodd (2016). "Delivery of Dietary and Lifestyle Interventions in Pregnancy: is it Time to Promote the Use of Electronic and Mobile Health Technologies?" Semin Reprod Med **34**(2): e22-27.

Olson, C. M., M. L. Graham, M. M. Demment, D. Fernandez, J. Reschke and S. Groth (2013). "Pregnant women use an online healthy weight gain program as designed." FASEB Journal **27**.

Peter, J. E., P. Barron and Y. Pillay (2015). "Using mobile technology to improve maternal, child and youth health and treatment of HIV patients." S Afr Med J **106**(1): 3-4.

Ramallo-Farina, Y., L. Garcia-Perez, I. Castilla-Rodriguez, L. Perestelo-Perez, A. M. Wagner, P. de Pablos-Velasco, A. C. Dominguez, M. B. Cortes, L. Vallejo-Torres, M. E. Ramirez, P. P. Martin, I. Garcia-Puente, M. A. Salinero-Fort and P. G. Serrano-Aguilar (2015). "Effectiveness and cost-effectiveness of knowledge transfer and behavior modification interventions in type 2 diabetes mellitus patients--the INDICA study: a cluster randomized controlled trial." Implement Sci **10**: 47.

Rotheram-Borus, M. J., M. Tomlinson, D. Swendeman, A. Lee and E. Jones (2012). "Standardized functions for smartphone applications: examples from maternal and child health." Int J Telemed Appl **2012**: 973237.

Schuster, N. (2014). "Health apps: Mothered by smartphone." Pharmazeutische Zeitung(28).

Stabile, M. G. (2015). "The granting of the phone, that is a headstrong mother, a stubborn pediatrician." Medico e Bambino **34**(1): 50-51.

Stevenson, S. J., A. M. Wojcieszek, F. Boyle, S. Vlack, D. Ellwood, G. Gardener and V. Flenady (2016). "My baby's movements: developing mobile phone software to increase maternal awareness of fetal movements." Journal of Paediatrics and Child Health **52**: 17-18.

Suleman, Z. (2015). "Journey of a thousand miles: harnessing mobile communications technology to solve problems in maternal health and child mortality in Balochistan, Pakistan." IEEE Pulse **6**(1): 28-31.

Takeuchi, S. and S. Horiuchi (2016). "Randomised controlled trial using smartphone website vs leaflet to support antenatal perineal massage practice for pregnant women." Women Birth **29**(5): 430-435.

Tombor, I., L. Shahab, J. Brown, D. Crane, S. Michie and R. West (2016). "Development of SmokeFree Baby: a smoking cessation smartphone app for pregnant smokers." Transl Behav Med **6**(4): 533-545.

Wang, H. J. and I. O. Kim (2015). "[Effects of a Mobile Web-based Pregnancy Health Care Educational Program for Mothers at an Advanced Maternal Age]." J Korean Acad Nurs **45**(3): 337-346.

Zairina, E., M. J. Abramson, C. F. McDonald, J. Li, T. Dharmasiri, K. Stewart, S. P. Walker, E. Paul and J. George (2015). "Study protocol for a randomised controlled trial evaluating the efficacy of a telehealth program--management of asthma with supportive telehealth of respiratory function in pregnancy (MASTERY(c))." BMC Pulm Med **15**: 84.
