# Supplementary material for: The Effect of Mobile App Interventions on Influencing Healthy Maternal Behavior and Improving Perinatal Health Outcomes: Systematic Review
Source: JMIR Mhealth Uhealth. 2018 Aug 9;6(8):e10012. doi: 10.2196/10012 (PMC6107729; doi:10.2196/10012)
Supplement: Multimedia Appendix 3 [file mhealth_v6i8e10012_app3.pdf]

Multimedia Appendix 3: Bias assessment

	Study Author (Year)							
	**Ainscough (2016)**		**Choi (2016)**		**Ledford (2016)**		**Zairina (2016)**	
**Bias**	**Risk**	**Support**	**Risk**	**Support**	**Risk**	**Support**	**Risk**	**Support**
**Random sequence generation (selection bias)**	Unclear	Not described.	Low	Computer-generated randomization, stratified by self-reported pre-pregnancy BMI category.	Low	Pre-assigned block randomisation (each block n=40).	Low	Computer-generated allocation in a ratio of 1:1 for intervention or controls, in random blocks of four and six.
**Allocation concealment (selection bias)**	Unclear	Not described.	Low	Allocation concealed in opaque envelopes.	Unclear	Not described if those assigning participants to intervention groups were blinded.	Low	Allocation concealed in opaque envelopes.
**Blinding of participants and personnel (performance bias)**	High	Participants not blinded to intervention. Unknown whether researchers blinded.	High	Blinding participants and research staff was not possible due to nature of the intervention.	High	Providers blinded to patient participation in study. Impossible to blind participants to intervention group.	High	Blinding participants and research staff was not possible due to nature of the intervention.
**Blinding of outcome assessors (detection bias)**	Unclear	Not described.	High	Participants and researchers aware of group assignment.	Unclear	Data was analysed separately to data collection. Blinding not specified.	Low	Outcome assessments performed by research assistants masked to group allocation.
**Incomplete outcome data (attrition bias)**	Unclear	Not described.	Low	Low dropout rate in intervention group; reasons not stated.	Low	No significant group differences detected. Reasons reported for attrition and exclusions.	Low	Intervention had 3 withdrawals/loss to follow-up from 3 months. Controls had 1 withdrawal at 6-month follow-up.
**Selective reporting (reporting bias)**	Unclear	Not fully described. Abstract reports on one primary outcome.	Low	Authors reported results of primary and secondary outcomes.	Low	Authors reported outcomes of 3 primary hypotheses.	Low	Authors reported results of primary and secondary outcomes.
**Other sources of bias**	Unclear	Not described.	Unclear	Not described.	Unclear	Not described.	Unclear	Acknowledgements include in-kind support from Vitalograph Inc, manufacturers of COPD-6.

Study (publication year)	Random sequence generation	Allocation concealment	Blinding of participants & personnel	Blinding of outcome assessors	Incomplete outcome data	Selective reporting	Other bias
	**Risk assessment**						
Ainscough et al. (2016)	Unclear	Unclear	High	Unclear	Unclear	Unclear	Unclear
Choi et al. (2016)	Low	Low	High	High	Low	Low	Unclear
Ledford et al. (2016)	Low	Unclear	High	Unclear	Low	Low	Unclear
Zairina et al. (2016)	Low	Low	High	Low	Low	Low	Unclear
