# Supplementary material for: The Effect of Mobile App Interventions on Influencing Healthy Maternal Behavior and Improving Perinatal Health Outcomes: Systematic Review
Source: JMIR Mhealth Uhealth. 2018 Aug 9;6(8):e10012. doi: 10.2196/10012 (PMC6107729; doi:10.2196/10012)
Supplement: Multimedia Appendix 4 [file mhealth_v6i8e10012_app4.pdf]

Multimedia Appendix 4: Intervention characteristics

Study	Ainscough 2016	Zairina 2016	Ledford 2016	Choi 2016
**Participant risk category**	**Moderate**		**Low**	
**Domain**				
**User experience**				
**User or "pilot" testing of app in design or development stage**	-	-	-	Yes
**Links to shared participant "chat" spaces**	-	No	No	No
**Content**				
**App developed specifically for the intervention**	-	Yes	Yes	Yes
**App is publicly available**	-	No	No	No
**Study cites stakeholder review for quality assurance**	-	-	-	-
**Study cites evidence base for app content (e.g. references clinical practice guidelines, endorsed educational content, published manuals, etc)**	-	Yes	-	Yes
**Patient-provider communication**				
**Facilitation within the app by expert, clinician, other**	-	No	No	No
**Healthcare provider access to user data for tracking purposes**	-	Yes	-	No
**Functionality**				
**App connects to a device built in to phone**	No	No	No	No
**App connects to a device through a "plug-in"**	No	Yes	No	Yes
**"Push communication" feature where messages sent directly to user (e.g. reminders to use the app, fill in a survey)**	-	Yes	-	Yes
**Study provides information comparing cost of app intervention with other communication modes**	No	No	No	No
**Data tracking**				
**Feature(s) for user to record own data**	-	Yes	Yes	Yes
**Feature(s) to automatically record user data**	-	Yes	Yes	Yes
**User access to data for tracking**	-	Yes	Yes	Yes
**App privacy policies or terms of use articulated**	-	-	-	Yes

Notes: - Not reported.
