# Supplementary material for: The Effect of Mobile App Interventions on Influencing Healthy Maternal Behavior and Improving Perinatal Health Outcomes: Systematic Review
Source: JMIR Mhealth Uhealth. 2018 Aug 9;6(8):e10012. doi: 10.2196/10012 (PMC6107729; doi:10.2196/10012)
Supplement: Multimedia Appendix 5 [file mhealth_v6i8e10012_app5.pdf]

**Multimedia Appendix 5: Inventories used in included studies**

| Inventory | Range of scores | Comment | Study author |
|---|---|---|---|
| Questionnaire developed from the Transtheoretical Model | Stage-of-change score (1-5) | Assesses readiness to engage in new or change existing lifestyle behaviours. | Ainscough |
| Stanford Brief Physical Activity Survey (SBAS) | 5 response choices per item, including a global statement about the activity and the dimensions of frequency, intensity, time, and type of activity. | 2-item, self-report PA assessment tool designed to obtain a quick assessment of the usual amount and intensity of physical activity that a person currently performs throughout the day. | Choi |
| Physical Activity Stages of Change | Scores motivational readiness for behaviour change (from pre-contemplation to maintenance stages) | 4-item questionnaire with Yes/No responses | Choi |
| Self-Efficacy for Physical Activity | 6 (low) to 30 (high) | 5-item questionnaire measuring stages of change for exercise behaviour | Choi |
| Social Support and Exercise Survey | 13 (less) to 65 (more support) | 13-item questionnaire, each with 5-point scale, listing things people might do or say to someone who is trying to exercise regularly. | Choi |
| Barriers to Being Active Quiz (CDC) | 0 (low) to 9 (high) | 21-item questionnaire on 4-point scale. Barriers to physical activity fall into 1 or more of 7 categories: lack of time, social influences, lack of energy, lack of willpower, fear of injury, lack of skill, and lack of resources. | Choi |
| Center for Epidemiological Studies Depression Scale (CES-D) | 0 (no) to 60 (severe depression) | 20-item questionnaire with 4 response categories, asking respondents how they may have felt or behaved. | Choi |
| Pregnancy Discomfort Checklist (modified) | Frequency, Severity: 32 (less) to 128 (more). Distress: 0 (less) to 128 (more) | Assessed the severity and “distressfulness” of symptoms in addition to frequency, sleep disturbance and restless legs syndrome. | Choi |
| Patient activation measure (PAM) | 0=not activated, 100=most activated | 13 Likert-type items to create a continuous patient activation measure. Done at baseline and 32 wk gestation appt. | Ledford |
| Prenatal Interpersonal Processes of Care (PIPC) scale | Scale of 0-100. Items averaged to create subscales. Subscales averaged to calculate overall PIPC score. | 30 items, each with 7-point scale. PIPC is conceptualized as the social and psychological aspects of provider interactions with patients. Focuses on 3 dimensions: Communication, Patient-Centered Decision Making, and Interpersonal Style. | Ledford |
| Asthma Control Questionnaire (ACQ-7) | ACQ score is the mean of the 7 items, 0 (well controlled) and 6 (extremely poorly controlled). | 7-item questionnaire, each with 7-point scale. Given at 3- and 6-months for primary outcome measures. Can be completed in the clinic without daily recordings of symptoms, medication use and airway calibre. Patients recall their experiences during the previous 7 days. | Zairina |
| Juniper’s mini-Asthma Quality-of-life Questionnaire (mAQLQ) | Score is the mean of all responses. | 15-item questionnaire, each with 7-point scale. Self-administered, with patients asked to recall their experiences during the previous 2 weeks. Measures 4 domains of symptoms, activity limitation, emotional function and environmental stimuli. | Zairina |

**References:**

Ainscough K, Kennelly M, Lindsay KL, O'Sullivan EJ, McAuliffe FM: Impact of an mHealth supported healthy lifestyle intervention on behavioural stage of change in overweight and obese pregnancy. Proceedings of the Nutrition Society 2016, 75.

Choi J, Lee JH, Vittinghoff E, Fukuoka Y: mHealth Physical Activity Intervention: A Randomized Pilot Study in Physically Inactive Pregnant Women. Matern Child Health J 2016, 20:1091-1101.

Ledford CJ, Canzona MR, Cafferty LA, Hodge JA: Mobile application as a prenatal education and engagement tool: A randomized controlled pilot. Patient Educ Couns 2016, 99:578-582.

Zairina E, Abramson MJ, McDonald CF, Li J, Dharmasiri T, Stewart K, Walker SP, Paul E, George J: Telehealth to improve asthma control in pregnancy: A randomized controlled trial. Respirology 2016, 21:867-874.
