# Supplementary material for: The Effect of Mobile App Interventions on Influencing Healthy Maternal Behavior and Improving Perinatal Health Outcomes: Systematic Review
Source: JMIR Mhealth Uhealth. 2018 Aug 9;6(8):e10012. doi: 10.2196/10012 (PMC6107729; doi:10.2196/10012)
Supplement: Multimedia Appendix 6 [file mhealth_v6i8e10012_app6.pdf]

Multimedia Appendix 6: Secondary outcomes

Two of the four trials reported secondary outcomes relevant to this review, with data provided below.

Outcomes	N	Intervention	N	Controls	*P*-value (where reported)
**Zairina et al. (2016)**					
**Major adverse maternal outcome (composite of death, admission to intensive care unit or near-miss mortality as defined by WHO)**					
Postpartum haemorrhage	33	1 (3%)	36	2 (6%)	NR
"Hypertensive disorders of pregnancy"	33	2 (6%)	36	2 (6%)	NR
Gestational diabetes mellitus	33	3 (9%)	36	6 (17%)	NR
Elective caesarean birth	33	6 (18%)	36	6 (17%)	NR
Emergency caesarean birth	33	4 (12%)	36	6 (17%)	NR
**Maternal general health (as defined by standardised measures such as general health questionnaires)**					
FEV_1 – 6 months from baseline	33	0.11 ± 0.06	36	0.07 ± 0.05	0.57[a]
FEV_1/FEV_6 – 6 months from baseline	33	1.53 ± 1.07	36	-0.56 ± 0.98	0.16[a]
Prescribed an oral corticosteroid	33	1 user	36	2 users	
**Maternal psychosocial outcomes, such as satisfaction, self-efficacy or anxiety (as measured by any validated, standard instrument)**					
mAQLQ – 6 months from baseline[e]	33	0.51 ± 0.16	36	-0.22 ± 0.15	0.002[b,c]
**Health service utilisation (antenatal care attendance, maternal antenatal admission, length of hospital stay of mother or infant)**					
Unscheduled health visit related to asthma	33	0 user	36	1 users	
**Choi et al. (2016)**					
**Maternal general health (as defined by standardised measures such as general health questionnaires)**					
**Pregnancy symptoms: severity**[f]	13	48.6±8.1	14	55.3±11.4	0.10[d]
**Pregnancy symptoms: frequency**[f]	13	67.1±13.7	15	73.7±13.7	0.17[d]
**Pregnancy symptoms: distress**[g]	13	70.0±17.6	14	73.4±17.7	0.91[d]
**Maternal psychosocial outcomes, such as satisfaction, self-efficacy or anxiety (as measured by any validated, standard instrument)**					
**Over the 12 weeks of follow-up, the intervention reduced perceived barriers to exercise including**[h]**:**					
Lack of time	13	3.08±2.25	15	4.13±2.26	0.08[d]
Social influence	13	2.23±2.49	15	2.73±1.34	0.21[d]
Lack of energy	13	3.62 ±2.90	15	4.80±2.08	0.02[c,d]

Lack of willpower	13	3.23±2.13	15	5.20±2.01	0.06[d]
Fear of injury	13	0.77±2.24	15	0.60±0.99	0.65[d]
Lack of skill	13	1.15±1.99	15	1.33±1.40	0.70[d]
Lack of resources	13	2.38±1.90	15	2.20±1.86	0.43[d]
**Self-efficacy at 12 weeks**[i]	13	18.7±4.4	15	17.1±5.2	0.58[d]
**Depression (CES-D) at 12 weeks**[j]	13	8.8±2.7	15	11.1±6.9	0.56[d]

Notes: Unless otherwise noted, values reported for intervention and controls groups are presented as mean ± standard error. [a]*P*-value of difference between groups, adjusted for baseline. [b]*P*-value of difference between groups adjusted for baseline. [c]Statistically significant. [d]Overall *P*-values based on ANCOVA models for changes from baseline to 12-week visit, adjusting for baseline. [e]Increased mAQLQ score suggests better asthma-related quality of life. [f]Possible scores from 32 (less) to 128 (more). [g]Possible scores from 0 (less) to 128 (more). [h]Possible scores from 0 (low) to 9 (high). [i]Possible scores from 6 (low) to 30 (high self-efficacy). [j]Possible scores from 0 (no) to 60 (severe depression). QoL improved from baseline. FEV_1=forced expiratory volume in 1 second. FEV_6=forced expiratory volume in 6 seconds. mAQLQ= Mini-Asthma quality of life questionnaire. N=number within sample reporting. NR=Not reported. QoL=quality of life. WHO=World Health Organization.

<u>References:</u>

Zairina E, Abramson MJ, McDonald CF, Li J, Dharmasiri T, Stewart K, et al. Telehealth to improve asthma control in pregnancy: A randomized controlled trial. *Respirology*. 2016 Jul;21(5):867-74. PubMed PMID: 27037722.

Choi J, Lee JH, Vittinghoff E, Fukuoka Y. mHealth Physical Activity Intervention: A Randomized Pilot Study in Physically Inactive Pregnant Women. *Maternal and child health journal*. 2016 May;20(5):1091-101. PubMed PMID: 26649879.
